# Hypertensive Emergency and Brainstem Hemorrhage Temporally Associated With Intramuscular Epinephrine: A Case Report

**DOI:** 10.7759/cureus.109136

**Published:** 2026-05-18

**Authors:** Kristine R Liang, Rhea Kundan, Trinity Puno, Nauman Tahir

**Affiliations:** 1 Anesthesiology, HCA Sunrise Health GME Consortium at Southern Hills Hospital, Las Vegas, USA; 2 Family Medicine, HCA Sunrise Health GME Consortium at Southern Hills Hospital, Las Vegas, USA; 3 General Surgery, College of Osteopathic Medicine, Rocky Vista University Montana, Billings, USA; 4 Nephrology, Southern Hills Hospital, Las Vegas, USA

**Keywords:** anaphylaxis, brainstem hemorrhage, epinephrine, hypertensive crisis, hypertensive emergency, intracranial hemorrhage

## Abstract

Epinephrine is the first-line therapy for anaphylaxis, and intramuscular (IM) administration at recommended doses is essential to prevent progression to airway compromise, circulatory collapse, and death. However, epinephrine is a potent sympathomimetic that can provoke severe cardiovascular responses in susceptible patients. We report the case of a 50-year-old African-American male with a history of long-standing hypertension, end-stage renal disease on intermittent hemodialysis with poor adherence, type two diabetes mellitus, and chronic hepatitis C, who developed acute neurological deterioration shortly after receiving two doses of 0.5 mg IM epinephrine for suspected anaphylaxis. Blood pressure measurements were unobtainable in the prehospital setting. In the emergency department, the patient was noted to have severe hypertension with an initial recorded blood pressure of 213/110 mmHg prior to intubation. Due to difficulty obtaining reliable noninvasive measurements thereafter, a radial arterial line was placed, revealing a blood pressure of 326/101 mmHg. Computed tomography (CT) of the brain demonstrated a large acute intraparenchymal hemorrhage involving the pons and midbrain with intraventricular extension. CT angiography excluded aneurysm or vascular malformation. Despite intensive care management and blood pressure control, the patient had limited neurological recovery and required prolonged ventilatory support, tracheostomy, and transfer to a long-term acute care hospital. This case highlights a rare but severe complication temporally associated with epinephrine, particularly in patients with underlying cerebrovascular risk factors. This case also underscores the importance of vigilant hemodynamic monitoring after epinephrine administration and early recognition of neurological deterioration.

## Introduction

Anaphylaxis is a systemic hypersensitivity reaction that may present rapidly, and it requires early recognition and immediate treatment with epinephrine to prevent progression to life-threatening complications, including airway compromise, circulatory collapse, shock, and death [1,2]. Intramuscular (IM) epinephrine is the cornerstone of anaphylaxis management because its alpha- and beta-adrenergic effects rapidly reverse bronchospasm, laryngeal edema, and circulatory collapse [2,3]. At standard IM doses (0.3-0.5mg for adults), epinephrine is generally safe; however, it may produce transient adverse effects such as anxiety, restlessness, headache, dizziness, palpitations, pallor, and tremor [2-6]. Nevertheless, its potent vasoconstrictive properties can rarely precipitate severe cardiovascular complications such as hypertensive crises, arrhythmias, myocardial ischemia, pulmonary edema, and, in extreme cases, intracranial hemorrhage (ICH) [2,7].

In this report, we present the case of a patient with multiple vascular risk factors who developed a large pontine and midbrain hemorrhage with intraventricular extension after receiving two standard doses of IM epinephrine for presumed anaphylaxis. To our knowledge, this is the ninth reported case of hypertensive ICH associated with epinephrine administration, adding to the limited body of literature on this rare but devastating complication. This case also represents an extremely elevated blood pressure compared with previously reported cases [8-15]. The highest blood pressure recorded amongst previously published hypertensive ICH case reports was 240/130 [13]. Awareness of this rare complication is essential to guide monitoring and clinical decisions after epinephrine administration.

## Case presentation

The patient is a 50-year-old African-American male with a history of hypertension, end-stage renal disease (ESRD) on hemodialysis three times weekly with poor adherence, type two diabetes mellitus, and chronic hepatitis C. His mother called emergency medical services (EMS) for symptoms of acute dyspnea, reported throat tightness, and inability to speak approximately 15-20 minutes after exposure to an ammonia-based glass cleaner. EMS documented stridor and administered two IM doses of 0.5 mg epinephrine along with 50 mg intravenous diphenhydramine for presumed anaphylaxis. The patient transiently improved but subsequently developed expressive aphasia, lethargy, and respiratory distress necessitating endotracheal intubation in the emergency department.

Prehospital blood pressure measurements were unobtainable. In the emergency department, the patient was noted to have severe hypertension with an initial recorded blood pressure of 213/110 mmHg prior to intubation. Subsequent noninvasive blood pressures were difficult to obtain reliably, prompting placement of a right radial arterial line, which revealed a blood pressure of 326/101 mmHg. Neurologic examination revealed obtundation, unequal pupils sluggish to light, and left-sided hemiplegia. Emergent noncontrast head computed tomography (CT) demonstrated a large acute intraparenchymal hemorrhage involving the dorsal midbrain and pons with intraventricular extension into the fourth ventricle (Figure 1). CT angiography of the head and neck excluded aneurysm, arteriovenous malformation, and large-vessel occlusion. Neurosurgery deemed the hemorrhage nonoperative.

**Figure 1:**
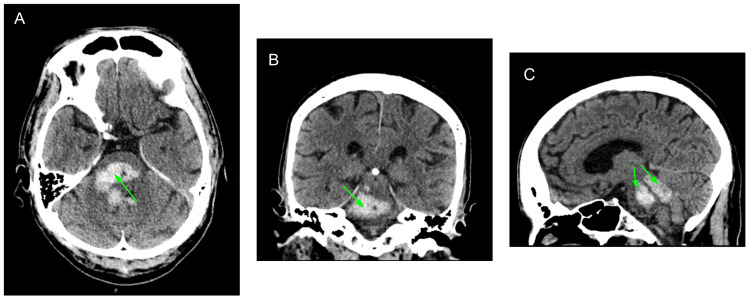
Initial noncontrast CT of the brain demonstrating a large intraparenchymal hemorrhage (green arrow) involving the dorsal midbrain and pons with intraventricular extension into the fourth ventricle in axial view (A), coronal view (B), and sagittal view (C). CT, computed tomography

The patient was admitted to the intensive care unit, where a nicardipine infusion was initiated for blood pressure control with a target systolic pressure of approximately 140 mmHg. Repeat CT head imaging within 24 hours showed stable brainstem hemorrhage. Magnetic resonance imaging of the brain without contrast confirmed the pontine and midbrain hemorrhages. Electroencephalogram revealed no seizure activity. Due to poor neurologic recovery, tracheostomy and percutaneous endoscopic gastrostomy were performed. The patient later demonstrated limited ability to follow commands, and, thus, he was discharged to a long-term acute care hospital after 14 days of hospitalization. He returned four days following discharge for altered mental status. Repeat CT head at his second admission showed evolution of the pontine hematoma with surrounding vasogenic edema and local mass effect (Figure 2).

**Figure 2:**
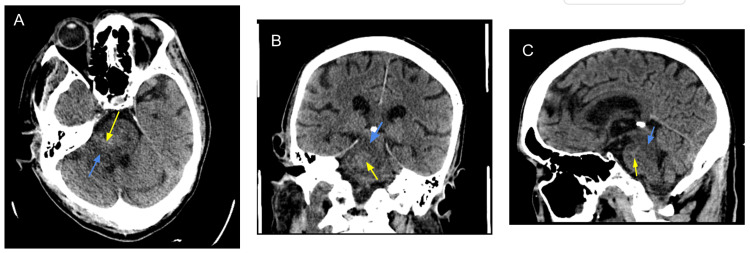
Follow-up CT brain four days after initial discharge (18 days after initial presentation), showing evolution of the pontine hematoma (yellow arrow) with surrounding vasogenic edema (blue arrow) and local mass effect in axial view (A), coronal view (B), and sagittal view (C). CT, computed tomography

## Discussion

Although IM epinephrine is widely regarded as safe, rare cases of hypertensive ICH following therapeutic doses have been documented [8-15]. Our patient’s presentation is consistent with previously reported cases: rapid neurologic decline within hours of epinephrine administration, hypertensive emergency, and ICH in deep brain structures that are vulnerable to hypertensive injury. The pathophysiology likely involves alpha-adrenergic vasoconstriction and beta-adrenergic cardiac stimulation, leading to marked increases in systemic vascular resistance and blood pressure [16]. In patients with chronic hypertension, ESRD, and cerebrovascular fragility, autoregulatory thresholds are easily exceeded, predisposing to vessel rupture [8].

In our patient, multiple risk factors, including poorly controlled hypertension, nonadherence with hemodialysis, and chronic vascular disease, likely contributed to the hemorrhage. The initial presentation cannot be confirmed with absolute certainty as anaphylaxis. The patient's symptoms of dyspnea, throat tightness, inability to speak, and EMS-documented stridor were clinically concerning for possible anaphylaxis at the time of EMS evaluation and appropriately prompted treatment with IM epinephrine. However, confirmatory diagnostic testing was not available, and alternative etiologies cannot be fully excluded retrospectively. Additionally, prehospital blood pressure measurements were unobtainable, but severe hypertension (213/110 mmHg) was documented initially, followed by markedly elevated blood pressure measurement of 326/101 mmHg. Although causality cannot be definitely established from this case, the temporal association between epinephrine administration, escalating blood pressures, and neurologic deterioration supports a possible association between epinephrine administration and hypertensive ICH in this highly susceptible patient.

The literature on this rare complication remains limited to isolated case reports. Yamamoto et al. described a dialysis patient who developed ICH after IM adrenaline for anaphylaxis [8]. Levis et al. reported prehospital IM epinephrine-associated ICH [9]. Several other reports document fatal or severe neurological outcomes following therapeutic epinephrine doses [10-15]. Despite these reports, the overwhelming evidence supports the safety of IM epinephrine in anaphylaxis [17]. The risk of untreated anaphylaxis far exceeds the rare possibility of hemorrhagic complications [2, 17]. Because anaphylaxis is immediately life-threatening, guidelines recommend prompt IM epinephrine when suspected, and these recommendations should not be interpreted as contraindications in most high-risk patients. Rather, this case highlights that in patients with multiple risk factors for hypertensive complications, medical personnel should rapidly reassess evolving signs after epinephrine administration, monitor hemodynamics closely, and consider alternative diagnoses when features are atypical. Early invasive blood pressure monitoring and aggressive but controlled antihypertensive therapy are essential when hypertensive emergencies are identified. The few prior reports of ICH associated with epinephrine use suggest that although the absolute risk is very low, the clinical consequence can be severe and potentially fatal. Therefore, reporting additional cases with careful timelines and risk factor data is important to inform risk stratification and management guidance.

## Conclusions

We describe a rare case of pontine and midbrain hemorrhage associated with therapeutic IM epinephrine in a patient with multiple vascular risk factors. Impaired vascular autoregulation from ESRD, chronic hypertension, and abrupt sympathetic stimulation may have contributed to severe hypertensive escalation and subsequent brainstem hemorrhage in this susceptible patient. Although epinephrine remains the first-line and life-saving treatment for anaphylaxis, medical personnel should be aware of this rare complication. Careful post-administration monitoring and consideration of underlying comorbidities may help identify at-risk patients and facilitate early recognition of adverse outcomes. Further reporting and investigation are warranted to better quantify risk and guide clinical practice.
